# Editorial: Model systems in hearing research

**DOI:** 10.3389/fcell.2024.1340344

**Published:** 2024-01-19

**Authors:** Emma J. Kenyon, Ben Warren, Rahul Mittal, Eri Hashino

**Affiliations:** ^1^ Swansea University Medical School, Swansea, United Kingdom; ^2^ Neurogenetics, Leicester, East Midlands, United Kingdom; ^3^ Department of Otolaryngology, Leonard M. Miller School of Medicine, University of Miami, Miami, FL, United States; ^4^ Department of Otolaryngology, Head and Neck Surgery, School of Medicine, Indiana University Bloomington, Indianapolis, IN, United States

**Keywords:** hearing, model system, editorial, animal, *in vivo*, *in vitro*

Researchers continue to be captivated by the challenge of understanding how hearing works. However, hearing research is fraught with obstacles - from the inaccessible bone-enclosed cochlea that operates at many kilohertz to complex neural networks in the brain that compute sub-millisecond latencies. Over the last half-decade, rapid progress has advanced our understanding of hearing, from the micromechanical process of transduction to the discovery of the sound-activated ion channel to the networks that compute auditory information. Along the way, step changes in our understanding have been fueled by diverse hearing models. Leading the way are mammalian systems, especially transgenic mice that recapitulate mutations found in human deafness. This Research Topic looks even more widely at less established model systems that also add to our understanding of hearing.

We use model systems throughout biology because the biological phenomenon is complex. There are many models used in hearing research. These include classical *in vivo* systems (mice, rats, zebrafish, fruit flies), non-classical *in vivo* systems (locusts), and *in vitro* systems (organoids and organotypic cochlea tissue cultures). With such a wide variety of systems, it is important to consider the advantages and limitations of each and their relevancy to human hearing. What aspect of hearing are we investigating? And to solve it, do we require a regenerative system, a genetically tractable system, or a physiologically amenable system? Many of the non-mammalian models have a substantially different ear anatomy to their mammalian counterparts; for example, only mammals have a coiled cochlea and a three-ossicle middle ear. Despite these differences, non-mammalian models have advantages in identifying therapies for hearing loss in humans. For example, fish and birds’ sensory hair cells have high regenerative capability, with many of the gene pathways shared with mammals. Zebrafish have also proved to be the model of choice when seeking a high throughput system for identifying otoprotective compounds or in identifying the ototoxic potential of new compounds. This is explored by Barrallo-Gimeno and Llorens in their review “*Hair cell toxicology: With the help of a little fish*”.

A number of studies have combined the *in vivo* advantages of the zebrafish model with an *in vitro* mouse model, such as organotypic cochlea tissue cultures, to identify potential otoprotective compounds. This should improve the probability of success when compounds are tested through *in vivo* mammalian assays. Derudas et al. used this combined approach in their article “*Charge and Lipophilicity are Required for Effective Block of the Hair-Cell Mechano- Electrical Transducer Channel by FM1-43 and its Derivatives*”. In this study, they identify the moieties required for a compound to load into hair cells or the moieties that effectively block the mechanoelectrical transduction (MET) channel. This gives insights into whether these can be altered to protect hair cells from ototoxins and poses questions about where exactly blockers of the hair cell MET complex bind within or relative to the hair cells ion conduction pathway.

The vestibular system has proved itself a valuable system to understand the basic principles of hearing. The Baeza-Loya and Raible review focuses on vestibular physiology in zebrafish. The zebrafish inner ear consists of the semi-circular canals, utricle, saccule, lagena, and macula neglecta, with the saccule regarded as the main hearing organ. The saccule has tonotopic organization, which provides key information on what physiological specialisms in hair cells lead to high-frequency hearing. The review also underscores the trackability of the neural circuits that process vestibular information. The zebrafish vestibular system is well placed to bridge the detection of vibrations by hair cells to behavior. The zebrafish not only has a more anciently evolved hearing system; its evolution in an aquatic environment as opposed to air imposes its own biophysical constraints, which provides an informative comparison to mammals.

More recently, insects have stepped forward to offer insights into the fundamental principles of hearing. The morphology of insect ears is not only different from those of mammals but even between other insects. Despite such obvious morphological differences, insect ears share common principles of operation, such as age-related auditory decline. In their article “*Metabolic decline in an insect ear: correlative or causative for age-related auditory decline?*”, Austin et al. exploit the rapidly aging nature of the desert locust and its physiologically amenable hearing organ to investigate if a decrease in ear metabolism can explain hearing decline with age. They show that although metabolism correlates with auditory decline, it is not the causative agent, and they hypothesize that age-related auditory decline is more likely due to accumulative damage in multiple cell types, many of which are outlined in a review by López-Otín et al., 2013.

The classic insect model *Drosophila* has been employed in the article by Requena et al. named “*A Drosophila model for Meniere’s disease: Dystrobrevin is required for support cell function in hearing and proprioception*” to investigate the genes involved in the complex and multifaceted hearing and balance disorder, Meniere’s disease. They investigate the dystrobrevin genes, identified in whole exome sequencing to be linked with Meniere’s disease, and show their expression in the fly’s equivalent of the inner ear. This disease is not only rare but also has a complex etiology. As no effective treatment modalities are yet available, advances in our fundamental understanding are vital to build toward therapies.

We often tend to think of hearing research synonymously with human deafness, but hearing research can have different human health implications, as outlined by Loh et al. in this Research Topic. Their review of the mosquito auditory system raises some interesting fundamental questions on hearing and has implications for stopping the spread of disease-transmitting mosquitos, thus reducing the prevalence of diseases such as malaria.

Finally, animal models have been fundamental to hearing research, primarily because the morphology and function of the ear are currently prohibitive to recapitulate in cell cultures. In particular, the function of the hair cell, the unit of hearing, has yet to be functionally recreated in a cell culture assay. Costa et al. in their article “*Repurposing the lineage-determining transcription factor Atoh1 without redistributing its genomic binding sites*” show that Atoh1 is repurposed from a neuronal determinant to hair cell factor by the combined activity of Gfi1 and Pou4f3. The findings of this study not only have implications for research on the regeneration of mammalian hair cells but also for making functional hair cell lines. Costa et al. lay crucial groundwork for the use of cell cultures in hearing research, and although hair cell culture work is still in its infancy, the therapeutic potential is immense.

In this Research Topic, we have showcased articles that use insects to understand the fundamental processes of hearing malfunction, reviews on zebrafish as a model, an article using zebrafish and mouse, and, finally, one using hair cell-like cell lines, each of these answering different questions in the auditory field. One of the most promising approaches to improve inherited and acquired hearing loss in humans is the genetic re-programming of cells either in the ear or the re-programming of cells in culture that can replace faulty cells within the ear. Fortunately, gene pathways that control the development and maintenance of hearing are highly conserved across hearing models. Due to this, gene pathways and interactions can be rapidly chartered at one end of the spectrum (e.g., insects), whilst the specificities of hair cell function and auditory processing can be chartered at the other (e.g., zebrafish). We believe that the newly emerging cell culture organoids and established mammalian hearing models (e.g., mice) provide a nexus point where both approaches can merge before translation to humans.

